# Adaptive Model Predictive Control for Mobile Robots with Localization Fluctuation Estimation

**DOI:** 10.3390/s23052501

**Published:** 2023-02-23

**Authors:** Jie Meng, Hanbiao Xiao, Liyu Jiang, Zhaozheng Hu, Liquan Jiang, Ning Jiang

**Affiliations:** 1 Intelligent Transportation Systems Research Center, Wuhan University of Technology, 1178 Heping Avenue, Wuhan 430000, China; 2 Chongqing Research Institute, Wuhan University of Technology, 598 Liangjiang Avenue, Chongqing 400000, China; 3 Hubei Institute of Measurement and Testing Technology, 2 Maodianshan Middle Road, Wuhan 430000, China; 4 The State Key Laboratory of New Textile Materials and Advanced Processing Technologies, Wuhan Textile University, 1 Yangguang Avenue, Wuhan 430000, China; 5 School of Mechanical Science and Engineering, Huazhong University of Science and Technology, 1037 Luoyu Road, Wuhan 430000, China

**Keywords:** model predictive control, mobile robots, localization fluctuations, fuzzy estimation

## Abstract

Mobile robots are widely employed in various fields to perform autonomous tasks. In dynamic scenarios, localization fluctuations are unavoidable and obvious. However, common controllers do not consider the impact of localization fluctuations, resulting in violent jittering or poor trajectory tracking of the mobile robot. For this reason, this paper proposes an adaptive model predictive control (MPC) with an accurate localization fluctuation assessment for mobile robots, which balances the contradiction between precision and calculation efficiency of mobile robot control. The distinctive features of the proposed MPC are three-fold: (1) Integrating variance and entropy—a localization fluctuation estimation relying on fuzzy logic rules is proposed to enhance the accuracy of the fluctuation assessment. (2) By using the Taylor expansion-based linearization method—a modified kinematics model that considers that the external disturbance of localization fluctuation is established to satisfy the iterative solution of the MPC method and reduce the computational burden. (3) An improved MPC with an adaptive adjustment of predictive step size according to localization fluctuation is proposed, which alleviates the disadvantage of a large amount of the MPC calculation and improves the stability of the control system in dynamic scenes. Finally, verification experiments of the real-life mobile robot are offered to verify the effectiveness of the presented MPC method. Additionally, compared with PID, the tracking distance and angle error of the proposed method decrease by 74.3% and 95.3%, respectively.

## 1. Introduction

Mobile robots are being progressively used in numerous scenarios such as unmanned factories, logistics centers, and exhibition halls, thanks to their superior flexibility and maneuverability [1,2,3]. For unmanned operations, autonomous navigation technology is intuitively important for robots [4]. To follow a given trajectory, mobile robots have to be able to control their pose precisely and robustly based on the localization results [5]. However, localization and control issues are often studied independently, leaving robot control performers to be improved.

The control system needs accurate localization results as a reference to maintain good trajectory-tracking accuracy [6]. In traditional control methods, kinematic or dynamic modelling or the control theory have been given more attention, and localization results are always seen as an absolute truth value [7,8]. However, in practice, mobile robots, whether using vision-based or LiDAR-based localization solutions, are subject to noise interference from their external sensors, which may lead to fluctuations in localization [9,10]. If the controller still treats the localization result as absolute truth, this can lead to severe jittering or large tracking deviations. *To this end, this paper solves the robust control problem of mobile robots under the localization fluctuations, including the accurate localization fluctuation estimation and the robust controller design*.

Through the above analysis, to improve the operation accuracy of the four-wheel differential mobile robot, this paper analyses the localization fluctuation state in the dynamic scene and then realizes the adaptive adjustment of the predictive step size of the model predictive control. Therefore, the operation adaptability of the four-wheel differential mobile robot is improved.

(1) Integrating variance and information entropy—an enhanced localization fluctuation estimation method based on fuzzy logic rules is proposed to improve the accuracy of the fluctuation assessment.
(2) A modified kinematics model with external disturbance using the Taylor expansion-based linearization is established, which is convenient for controller design under localization fluctuations.
(3) An improved MPC with an adaptive adjustment of predictive step size related to localization fluctuation is proposed, which ensures the stability of the control system in dynamic scenes.
(4) The proposed method has been tested in real dynamic scenarios and compared with mainstream methods, and its effectiveness has been demonstrated.

## 2. Related Works

### 2.1. Localization Fluctuation Estimation

The problem of localization can be divided into simultaneous localization and map building (SLAM) and localization based on an a priori map, depending on the presence or absence of an a priori map [11]. Mainstream SLAM methods such as ORB-SLAM [12] and LEGO-LOAM [13] can generate a map of the environment while obtaining localization results. However, this method is subject to cumulative errors and has poor real-time performance. In contrast, a priori map-based localization provides accurate and efficient positional information and is often used as a reference for control [11]. Bayesian filter-based localization frameworks are currently the dominant approach using a priori map, such as Kalman filtering or Monte Carlo localization (MCL) [14,15,16]. In particular, MCL is widely used due to its ability to adapt to non-Gaussian non-linear scenarios [14,17]. Although there is much research around the robustness of MCL, existing algorithms are not immune to localization fluctuations in highly dynamic scenarios. The accurate description of the localization fluctuations is of great significance for the design of subsequent navigation systems. For this reason, Zapata et al. propose to use the maximum particle weight in the MCL as a benchmark to determine the current localization reliability [18]. When the maximum particle weight is less than the weight threshold, this is an indication that the currently estimated pose is not reliable. Nevertheless, it is not robust to use the weight of only one particle to measure localization reliability. In turn, variance and entropy values are common in addition to valid metrics for estimating localization fluctuations, which take into account the set of particles with weights in an integrated manner [19,20,21]. The variance is well understood mathematically and physically—the larger the variance, the greater the localization fluctuation—but is poorly described for localization data with multi-peaked distributions. Higher information entropy indicates smaller differences in particle weights, demonstrating greater uncertainty in localization. In particular, entropy is more accurate for non-convex data evaluations [22,23]. However, relying solely on a numerical metric is prone to misclassification. *In this regard, the main objective of this paper is to design a robust assessment method that integrates more localization fluctuation metrics into consideration*.

### 2.2. Mobile Robot Control

As one of the key modules, control technology has a great impact on the stability and accuracy of mobile robots [24]. In the process of motion, it is usually subject to external disturbances such as model uncertainty and parameter perturbation, resulting in motion oscillation and deviation, and even skidding and rollover [25]. To suppress disturbances, many scholars have made efforts to improve the performance of mobile bots. For example, the literature [26] designs a sliding mode control (SMC) scheme based on a reduced-order-extended-state observer, which realizes the active compensation of friction under the condition of uncertain parameters and ensures the stability of an operation. Through the smooth fitting of the path and design of MPC, the literature [27] realizes the high-speed movement of the four-wheeled independently steering robot with action delay. For the tracking problem of wheeled mobile robots with bounded disturbances and various practical constraints, a robust MPC method is proposed to ensure the safety and comfort during an operation [28]. At present, SMC and MPC have been the focus of research because of their excellent characteristics in high-speed and high-precision motion control methods [6,29,30]. Although SMC has the advantage of being insensitive to disturbances, the oscillation produced by itself is difficult to be eliminated, which makes it difficult to be widely used [31]. The gradual iterative optimization is brought by MPC, which can well handle the model constraints caused by structure, dynamic system, etc., and is conducive to achieving smooth motion [32,33]. This advantage improves the robustness of the control. In the design process of existing MPC methods, the observed localization data are usually treated as accurate values, which can ensure the stability of motion in static- or high-localization accuracy scenes [34,35]. Therefore, in the dynamic scene, the design of the controller needs to take into account the localization fluctuations, so as to avoid causing motion oscillation. *For this purpose, how to improve the robustness and accuracy of the controller in the localization fluctuation scenario has become a key issue to be studied in our work*.

## 3. System Modelling and Problem Formulation

### 3.1. System Modelling

**Figure 1:**
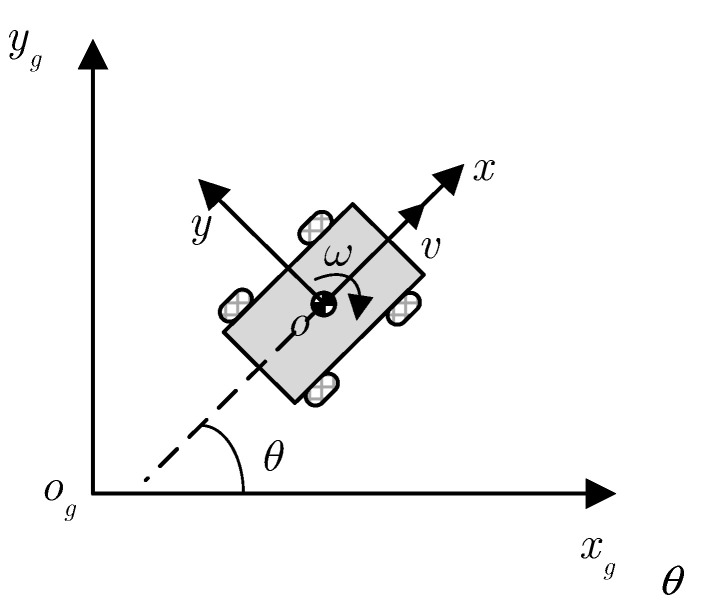
The four-wheel model and single-track model of the considered IWMD-MR.

Figure 1 shows the four-wheel differential platform model. The four-wheel differential platform has good motion performance and can achieve zero radius turning by adjusting the speed of the left and right wheels, which improves its adaptability to complex scenes. As the special case of mobile robots, the general modelling method can improve the general adaptability of the model [36,37]. Therefore, to further analyze the four-wheel differential platform and improve its motion controllability, the following general kinematics model of a mobile robot is given:

(1) χ=f(χ,u)

where $\chi ={\left[x,y,\theta \right]}^{T}$ is the state variable; $u={\left[v,\omega \right]}^{T}$ represents the control variable, *x* and *y* are the position of the mobile robot center point in the global fixed coordinate system, and θ denotes the robot heading angle; v and ω are the linear velocity and angular velocity of the mobile robot, respectively. The Taylor formula is used to expand the nonlinear mobile robot model at the reference point $\left(\chi_{r},u_{r}\right)$ to obtain the linear model of the mobile robot, so that the modelling accuracy can be guaranteed. 

At the same time, the linear model reduces the amount of computation, convenient for the design of the controller and is conducive to the actual implementation. Furthermore, at the Taylor expansion for Equation (1) at the point $\left(\chi_{r},u_{r}\right)$, we have:

(2) $$\dot{\chi }=f(\chi_{r},u_{r})+f_{\chi_{r}}(\chi -\chi_{r})+f_{u_{r}}(u-u_{r})+O_{r}$$

where $\chi_{r}$ is the reference control input; $u_{r}$ denotes the calculated reference input, $f_{\chi_{r}}(\cdot )$ and are coefficient matrices of function f(⋅) expanded at $\left(\chi_{r},u_{r}\right)$; O(r) is the higher order remainder of the Taylor expansion. By defining $e=\chi -\chi_{r}$, we have

(3) $$\dot{e}=f_{\chi_{r}}e+f_{u_{r}}\tilde{u}+O_{r}$$

where e represents the following error of the mobile robot; $\tilde{u}=u-u_{r}$ is the change of input control law. In the actual motion process, it is difficult to realize the continuous control of the mobile robot. Therefore, obtaining a discrete-time model of the four-wheel mobile robot is necessary. Setting the sampling period as T, where

(4) $$e(k+1)=e(k)+T\dot{e}(k)$$

where k is the sampling time. The above model is rewritten as

(5) $$e(k+1)=A(k)e(k)+B(k)\tilde{u}(k)+O_{r}(k)$$

where,

(6) $$A(k)=\left[\begin{array}{ccc} 1 & 0 & -T\cdot v_{r}\sin\theta_{r}(k)\\ 0 & 1 & T\cdot v_{r}\cos\theta_{r}(k)\\ 0 & 0 & 1 \end{array}\right],B(k)=\left[\begin{array}{cc} T\cos\theta_{r}(k) & 0\\ T\sin\theta_{r}(k) & 0\\ 0 & T \end{array}\right]$$

Therefore, in order to improve the controllability of the four-wheel mobile robot, we realized the linear modelling of the mobile robot kinematics model. This facilitates the design of the controller and makes the control of the mobile robot simpler.

### 3.2. Localization Problem Formulation

The mobile robot localization problem is often regarded as a typical Bayesian estimation problem. Bayesian filter-based localization algorithms solve robot localization problems by estimating the probability distribution of robot poses in the pose space and assigning a probability to each possible hypothetical pose using a confidence level (*Belief*), which is expressed as

(7) $$B(s_{k})=p(s_{k}|o_{1:k},u_{1:k},M)$$

where $s_{k}$ is the robot’s pose in the two-dimensional plane at time k, which can be expressed as $\left(x_{k},y_{k},\theta_{k}\right)$; $x_{k}$ and $y_{k}$ are the robot’s position, and $\theta_{k}$ is the robot’s heading; $o_{1:k}$ and $u_{1:k}$ represent the sensor observations and motion control from the initial time to time k, respectively; M is the a priori map.

According to the Markov hypothesis and Bayes rule, the Bayesian filter-based localization method can recursively estimate the robot’s poses, but it involves a large number of integral operations and nonlinear non-Gaussian features of the observation and motion models, which make the efficient and accurate solution a concern. To this end, this paper uses the MCL approach to compute Equation (7), which uses a particle set with weights to represent $B(s_{k})$, i.e.,

(8) $$B(s_{k})\;\approx \sum_{n_{p}=1}^{N_{p}}\omega_{k}^{\left[n_{p}\right]}\delta (s_{k}-s_{k}^{\left[n_{p}\right]})$$

where the set of particles with weights is denoted as ${\left\{\langle s_{k}^{\left[n_{p}\right]},\omega_{k}^{\left[n_{p}\right]}\rangle \right\}}_{n_{p}=1}^{N_{p}}$; $\omega_{k}^{\left[n_{p}\right]}$ is the weight of the $n_{p}$-*th* particle $s_{k}^{\left[n_{p}\right]}$ and $N_{p}$ means the total number of particles; $s_{k}^{\left[n_{p}\right]}$ can be written as $\left(x_{k}^{\left[n_{p}\right]},y_{k}^{\left[n_{p}\right]},\theta_{k}^{\left[n_{p}\right]}\right)$; $x_{k}^{\left[n_{p}\right]}$, $y_{k}^{\left[n_{p}\right]}$ and $\theta_{k}^{\left[n_{p}\right]}$ are the position and orientation of the particle; δ(·) is the Dirichlet function. Usually, the particle with the highest weight is selected as the current localization result.

## 4. Localization Fluctuations Estimation

Both variance and information entropy have their own unique advantages in expressing localization fluctuations and can reflect the characteristics of the particle set. However, a single performance metric still has a large randomness that affects the accuracy of the fluctuation assessment. To solve the above problems, a fuzzy logic rule incorporating variance and entropy is proposed to evaluate the localization fluctuations. Localization fluctuations are represented as follows:

(9) $$L_{f}={\left[L_{fx},L_{fy},L_{f\theta }\right]}^{T}={\left[f_{Vx}(V_{x})+f_{E}(E),f_{Vy}(V_{y})+f_{E}(E),f_{V\theta }(V_{\theta })+f_{E}(E)\right]}^{T}$$

(10) $$f_{Vi}(V_{i})=\left\{\begin{array}{cc} \alpha_{V} & 0\leq V_{i}<\eta_{1}V_{Ti}\\ \beta_{V} & \eta_{1}V_{Ti}\leq V_{i}<\eta_{2}V_{Ti}\\ \lambda_{V} & \eta_{2}V_{Ti}\leq V_{i} \end{array}\right.,\;f_{E}(E)=\left\{\begin{array}{cc} \alpha_{E} & 0\leq E<\eta_{3}E_{T}\\ \beta_{E} & \eta_{3}E_{T}\leq E<\eta_{4}E_{T}\\ \lambda_{E} & \eta_{4}E_{T}\leq E \end{array}\right.$$

where $V_{i}$ and E are the variance and entropy, respectively; i=x,y,θ; $f_{Vi}(V_{i})$ and $f_{Ei}(E_{i})$ are the localization fluctuation factors based on $V_{i}$ and E; $\alpha_{V}$, $\beta_{V}$, $\lambda_{V}$, $\alpha_{E}$, $\beta_{E}$ and $\lambda_{E}$ are the fluctuation parameters; $0<\alpha_{V}<\beta_{V}<\lambda_{V}$ and $0<\alpha_{E}<\beta_{E}<\lambda_{E}$; $\eta_{1}$, $\eta_{2}$, $\eta_{3}$ and $\eta_{4}$ are the weight coefficient; $0<\eta_{1}<\eta_{2}$ and $0<\eta_{3}<\eta_{4}$; $V_{Ti}$ and $E_{T}$ are the variance and entropy threshold value, severally. $L_{f}$ is a three-dimensional vector ${\left[L_{fx},L_{fy},L_{f\theta }\right]}^{T}$ that represents the fluctuations of *x*, *y*, and θ. Meanwhile, $L_{f}$ is determined by the variance and entropy values, where the entropy values are only related to the particle weights, so all three dimensions are set uniformly.

It is worth stating that the calculation of $V_{i}$ and E usually requires a series of localization results over a period of time. However, it is not practical to perform a large number of localization experiments in situ to determine the current localization fluctuation state, and we would prefer to conduct the evaluation depending on the localization data at a certain time. Fortunately, the MCL result is expressed as a particle set ${\left\{\langle s_{k}^{\left[n_{p}\right]},\omega_{k}^{\left[n_{p}\right]}\rangle \right\}}_{n_{p}=1}^{N_{p}}$, so that $V_{i}$ and E can be represented as

(11) $$V_{px}=\sum_{i=1}^{N_{p}}\omega_{k}^{\left[n_{p}\right]}{\left(x_{k}^{n_{p}}-{\bar{x}}_{k}^{}\right)}^{2},\;V_{py}=\sum_{i=1}^{N_{p}}\omega_{k}^{\left[n_{p}\right]}{\left(y_{k}^{n_{p}}-{\bar{y}}_{k}^{}\right)}^{2},\;V_{p\theta }=\sum_{i=1}^{n}\omega_{t}^{i}{\left(\theta_{t}^{i}-{\bar{\theta }}_{t}^{}\right)}^{2}$$

(12) $$E_{p}=-\sum_{n_{p}=1}^{N_{p}}\omega_{k}^{\left[n_{p}\right]}\log\omega_{k}^{\left[n_{p}\right]}$$

where $V_{pi}$ and $E_{p}$ are the variance and entropy from ${\left\{\langle s_{k}^{\left[n_{p}\right]},\omega_{k}^{\left[n_{p}\right]}\rangle \right\}}_{n_{p}=1}^{N_{p}}$; $\bar{*}$ denotes the mean value of the related particle’s pose.

Although $V_{i}$ and E can be reflected by the localization results at a certain time based on ${\left\{\langle s_{k}^{\left[n_{p}\right]},\omega_{k}^{\left[n_{p}\right]}\rangle \right\}}_{n_{p}=1}^{N_{p}}$, the exact relationship between $V_{pi}$, $V_{i}$, $E_{p}$ and E are still difficult to be indicated analytically. Fuzzy logic rules are an effective means to infer an output based on input variables. Hence, we integrate $V_{pi}$ and $V_{i}$ into a fuzzy formula using the following fuzzy logic rules:

(13) $$\begin{array}{ccccc} \mathrm{rule}\;1:\;if & 0\leq V_{pi}\leq V_{pi1} & then\;f_{Vi1}=f_{Vi}(V_{i}) & s.t. & 0\leq V_{i}<\eta_{1}V_{Ti}\\ \mathrm{rule}\;2:\;if & V_{pi2}\leq V_{pi}\leq V_{pi3} & then\;f_{Vi2}=f_{Vi}(V_{i}) & s.t. & \eta_{1}V_{Ti}\leq V_{i}<\eta_{2}V_{Ti}\\ \mathrm{rule}\;3:\;if & V_{pi4}\leq V_{pi} & then\;f_{Vi3}=f_{Vi}(V_{i}) & s.t. & \eta_{2}V_{Ti}\leq V_{i} \end{array}$$

where $V_{pi1},V_{pi2},V_{pi3}$ and $V_{pi4}$ are fuzzy demarcation boundaries for $V_{pi}$; $f_{Vi1},f_{Vi3}$ and $f_{Vi3}$ are the fluctuation values based on variance.

Similarly, the fuzzy formula for describing the mapping relation between $E_{p}$ and E can be written as

(14) $$\begin{array}{ccccc} \mathrm{rule}\;1:\;if & 0\leq E_{p}\leq E_{p1} & then\;f_{E1}=f_{E}(E) & s.t. & 0\leq E<\eta_{3}E_{T}\\ \mathrm{rule}\;2:\;if & E_{p2}\leq E_{p}\leq E_{p3} & then\;f_{E2}=f_{E}(E) & s.t. & \eta_{3}E_{T}\leq E<\eta_{4}E_{T}\\ \mathrm{rule}\;3:\;if & E_{p4}\leq E_{p} & then\;f_{E3}=f_{E}(E) & s.t. & \eta_{4}E_{T}\leq E \end{array}$$

where $E_{p1},\;E_{p2},\;E_{p3}$ and $E_{p4}$ are fuzzy demarcation boundaries for $E_{p}$; $f_{E1},\;f_{E3}$ and $f_{E3}$ are the fluctuation values based on entropy.

For implementation, the boundaries $V_{pi1}$ to $V_{pi4}$ and $E_{p1}$ to $E_{p4}$ can be learned from a mass of variance and entropy of localization results in different dynamic environments. As the next step of a standard procedure of constructing a fuzzy logic system, defuzzification is achieved by the weighted average method. Additionally, the fluctuation factor $L_{f}$ is obtained as follows:

(15) $$L_{f}={\left[L_{fx},L_{fy},L_{f\theta }\right]}^{T}={\left[\frac{\sum_{m=1}^{r}\chi_{m}f_{Vxm}}{\sum_{m=1}^{r}\chi_{m}}+\frac{\sum_{m=1}^{r}\chi_{m}f_{Em}}{\sum_{m=1}^{r}\chi_{m}},\frac{\sum_{m=1}^{r}\chi_{m}f_{Vym}}{\sum_{m=1}^{r}\chi_{m}}+\frac{\sum_{m=1}^{r}\chi_{m}f_{Em}}{\sum_{m=1}^{r}\chi_{m}},\frac{\sum_{m=1}^{r}\chi_{m}f_{V\theta m}}{\sum_{m=1}^{r}\chi_{m}}+\frac{\sum_{m=1}^{r}\chi_{m}f_{Em}}{\sum_{m=1}^{r}\chi_{m}}\right]}^{T}$$

where *r* is the number of fuzzy rule bases; $\chi_{m}$ denotes the trigger strength of *m*-th rule. If the $V_{pi}$ or $E_{p}$ satisfies the fuzzy rule, then $\chi_{m}$ is 1. Otherwise, $\chi_{m}$ is set as 0.

## 5. Adaptive MPC Considering Localization Fluctuation

In order to realize the robust control of the four-wheel mobile robot, based on the state space discrete model of Equation (5), we construct the cost function required in the optimal control process

(16) $$J\left(e(k),\tilde{u}(k)\right)=\sum_{i=1}^{N_{p}}{\left[e(k+i|k)\right]}^{T}P\left[e(k+i|k)\right]+\sum_{i=0}^{N_{c}-1}{\left[\tilde{u}(k+i|k)\right]}^{T}R[\tilde{u}(k+i|k)]$$

where J(⋅) is the cost function; e(k+i|k) and $\tilde{u}(k+i|k)$ represent the error value and control fluctuation value of time k+1 predicted at time k, respectively; $N_{p}$ and $N_{c}$ are prediction and control horizon, respectively, and the value of the control horizon is not greater than the prediction horizon; P and R are the weight matrix. 

To ensure the feasibility of optimal prediction, the control variables of the prediction process are as follows:

(17) $$\begin{array}{l} e_{\min}\leq e(k)\leq e_{\max}\\ u_{\min}\leq u(k)\leq u_{\max}\\ {\tilde{u}}_{\min}\leq \tilde{u}(k)\leq {\tilde{u}}_{\max}\\ u_{\min}\leq u(k)+T\tilde{u}(k)\leq u_{\max}\\ O_{r\min}\leq O_{r}(k)\leq O_{r\max} \end{array}$$

where $e_{\min}$ and $e_{\max}$ are the minimum and maximum errors, respectively. $u_{\min}$ and $u_{\max}$ are the minimum and maximum values of the control law increment, respectively. $O_{r\min}$ and $O_{r\max}$ are the minimum and maximum perturbations, respectively. 

Considering the existence of localization fluctuation, to ensure the stability of operation, $N_{p}$ and $N_{c}$ need to be further adjusted as follows:

(18) $$\begin{array}{l} N_{p}=[k_{1}\max(L_{f})]+k_{p}\\ N_{c}=[k_{2}\max(L_{f})]+k_{c} \end{array}$$

where $L_{f}$ is the estimated localization fluctuation value obtained from Equation (9), max() is the maximum value function of a vector, $k_{1,2}$ is the adjustment coefficient. $k_{c},k_{p}\in N_{+}$ is the minimum adjustment coefficient. [X] is the maximum integer value not greater than X. Considering the constraint state of the predictive control method, there are $N_{c}\leq N_{p}$.

Furthermore, using the cost function of Equation (16), the following state equation of the prediction horizon is obtained:

(19) $$\bar{e}(k)=F(k)\bar{\tilde{u}}(k)+L(k)e(k)+G(k)\tilde{u}(k-1)+{\bar{O}}_{r}(k)$$

with

(20) $$\begin{array}{l} \bar{e}(k)={\left[e(k+1|k),\ldots ,e(k+N_{p}|k)\right]}^{T}\\ \bar{\tilde{u}}(k)={\left[\tilde{u}(k+1|k),\ldots ,\tilde{u}(k+N_{c}-1|k)\right]}^{T}\\ {\bar{O}}_{r}(k)={\left[O_{r}(k+1|k),\ldots ,O_{r}(k+N_{p}-1|k)\right]}^{T} \end{array}$$

The coefficient matrix is expressed as:

(21) $$F(k)=\left[\begin{array}{ccc} B(k) & \ldots & 0\\ A(k+1)B(k) & \ldots & 0\\ \vdots & \ddots & \vdots \\ B(k+N_{p}-1)+\prod_{i=k+1}^{i=k+N_{p}-1}A(i)B(k) & \ldots & B(k+N_{p}-1)+\prod_{i=k+1}^{i=k+N_{p}-1}A(i)B(k+N_{c}) \end{array}\right]$$

(22) $$L(k)={\left[A(k),A(k+1)A(k),\ldots ,\prod_{i=k}^{i=k+N_{p}-1}A(i)\right]}^{T}$$

(23) $$G(k)=\left[\begin{array}{c} B(k)\\ B(k+1)+A(k+1)B(k)\\ \vdots \\ B(k+N_{p}-1)+\ldots +\prod_{i=k+1}^{i=k+N_{p}-1}A(i)B(k) \end{array}\right]$$

Therefore, in order to ensure the optimal operation process, the following optimization objectives are obtained by combining Formulas (16), (17) and (19):

(24) $$\min{\bar{e}}^{T}(k)P\bar{e}(k)+{\bar{\tilde{u}}}^{T}(k)R\bar{\tilde{u}}(k)$$

Considering modelling error and disturbance, Equation (19) is introduced into the optimization objective (24), thus:

(25) $$\min{\bar{O}}_{r}{}^{T}(k)P{\bar{O}}_{r}(k)+{\bar{\tilde{u}}}^{T}(k)R\bar{\tilde{u}}(k)$$

subject to

(26) $$\begin{array}{c} \min(e_{\max}-M(k),{\bar{O}}_{r\max})\leq {\bar{O}}_{r}(k)\leq \max(e_{\min}-M(k),{\bar{O}}_{r\min})\\ u_{\min}\leq u(k)\leq u_{\max}\\ {\tilde{u}}_{\min}\leq \tilde{u}(k)\leq {\tilde{u}}_{\max}\\ \;\;\;\;u_{\min}\leq u(k)+T\tilde{u}(k)\leq u_{\max} \end{array}$$

where $M(k)=F(k)\bar{\tilde{u}}(k)L(k)e(k)-G(k)\tilde{u}(k-1)$ is the intermediate variable.

By adjusting the above equation, the control law optimization under the scenario of disturbance fluctuation is realized.

Furthermore, the stability proof of the designed controller is given as follows:

**Theorem** **1.** *When the optimal control strategy of (19)–(25) is adopted and the following conditions are satisfied:*

(27) $$e(k+N_{p}|k)=0$$

(28) $$\tilde{u}(k+N_{c})=0$$

*Then, the system will approach stability.*

**Proof.**  The optimization function of the system can be rewritten as:

(29) $$\begin{array}{c} J\left(e(k+1),\tilde{u}(k+1)\right)=\sum_{i=1}^{N_{p}}{\left[e(k+i+1|k+1)\right]}^{T}P\left[e(k+i+1|k+1)\right]\\ \;\;\;\;\;\;\;\;\;\;+\sum_{i=0}^{N_{c}-1}{\left[\tilde{u}(k+i+1|k+1)\right]}^{T}R\left[\tilde{u}(k+i+1|k+1)\right]\\ \;\;\;\;\;\;\;\;\;\;\;\;\;\;\;\;\;\;\;\;\;\;\;=\sum_{i=1}^{N_{p}}{\left[e*(k+i+1|k)\right]}^{T}P\left[e*(k+i+1|k)\right]+\sum_{i=0}^{N_{c}-1}{\left[\tilde{u}*(k+i+1|k)\right]}^{T}R\left[\tilde{u}(k+i+1|k)\right] \end{array}$$

Combined with Formulas (27) and (28), there is

(30) $$\begin{array}{c} J\left(e(k+1),\tilde{u}(k+1)\right)=\sum_{i=1}^{N_{p}}{\left[e*(k+i|k)\right]}^{T}P\left[e*(k+i|k)\right]+\sum_{i=0}^{N_{c}-1}{\left[\tilde{u}*(k+i|k)\right]}^{T}R\left[\tilde{u}*(k+i|k)\right]\\ \;\;\;\;-{\left[e*(k+1|k)\right]}^{T}P\left[e*(k+1|k)\right]-{\left[\tilde{u}*(k|k)\right]}^{T}R\left[\tilde{u}*(k|k)\right]\\ \;\;\;\;\;\;\;\;\;=J*\left(e(k),\tilde{u}(k)\right)-{\left[e*(k+1|k)\right]}^{T}P\left[e*(k+1|k)\right]-{\left[\tilde{u}*(k|k)\right]}^{T}R\left[\tilde{u}*(k|k)\right] \end{array}$$

with $J^{*}\left(e(k+1),\tilde{u}(k+1)\right)$, which is the optimal control value at time k. Then, $e^{*}(k+i|k)$ and ${\tilde{u}}^{*}(k+i|k)$ are the predicted values obtained by solving the optimization function at time k to k+i. Hence, combining Equations (27) and (28), we have:

(31) $$e^{*}(k+1)=e^{*}(k+1|k),\;{\tilde{u}}^{*}(k)={\tilde{u}}^{*}(k|k)$$

Then,

(32) $$\begin{array}{cc} J^{*}\left(e(k+1),\tilde{u}(k+1)\right) & \leq J\left(e(k+1),\tilde{u}(k+1)\right)\\ & =J*\left(e(k),\tilde{u}(k)\right)-{\left[e*(k+1)\right]}^{T}P[e*(k+1)]-{\left[\tilde{u}(k)\right]}^{T}R[{\tilde{u}}^{*}(k)]\\ & \leq J^{*}\left(e(k),\tilde{u}(k)\right) \end{array}$$

Therefore, the optimal value of the cost function will gradually decrease in the process of iterative updating. It shows that the optimization method can guarantee the asymptotic convergence of the system. □

## 6. Experimental Validations 

### 6.1. Experimental Implementation

**Figure 2:**
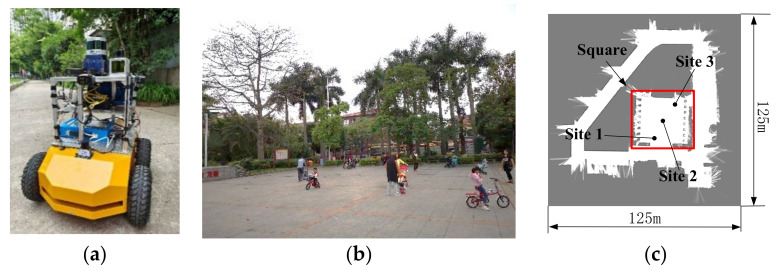
Experimental scene and platform. (**a**) Platform prototype. (**b**) Dynamic scene. (**c**) Grid map of the scene.

The experimental scenes and four-wheel differentially driven mobile robot are shown in Figure 2. The mobile robot basically consists of an industrial computer (Intel(R) Core (TM) i7-6500U CPU @2.50 GHz, 8 GB of RAM, 64-bit operating system), two LiDARs, four motor encoders and some related sensors, such as an ultrasonic transducer and anti-collision strip. More specifically, two UTM-30LX 2-D LiDARs with a range of 30 m and scanning rate of 40 Hz are used, which guarantee that the robot has enough field of view to ensure safety and real-time pose tracking. To avoid the blind spot of LiDAR, we installed four MaxBotix MB7360 ultrasonic sensors around the robot with a high resolution of 1 mm and a measuring distance of 5 m, which is sufficient to detect possible dynamic obstacles during the robot’s movement. As demonstrated in Figure 2b, the experimental environment is a dynamic and wide square surrounded by overgrown grass and a large number of pedestrians. Three sites are selected for the localization experiments to determine the fuzzy rule parameters. To comprehensively demonstrate the performance of the proposed control system, we first conduct localization experiments in different dynamic environments. Then, through real control experiments, the effectiveness of the proposed MPC method is verified.

### 6.2. Experimental Results and Discussions

#### 6.2.1. Experimental Results of Localization Fluctuation Estimation

**Figure 3:**
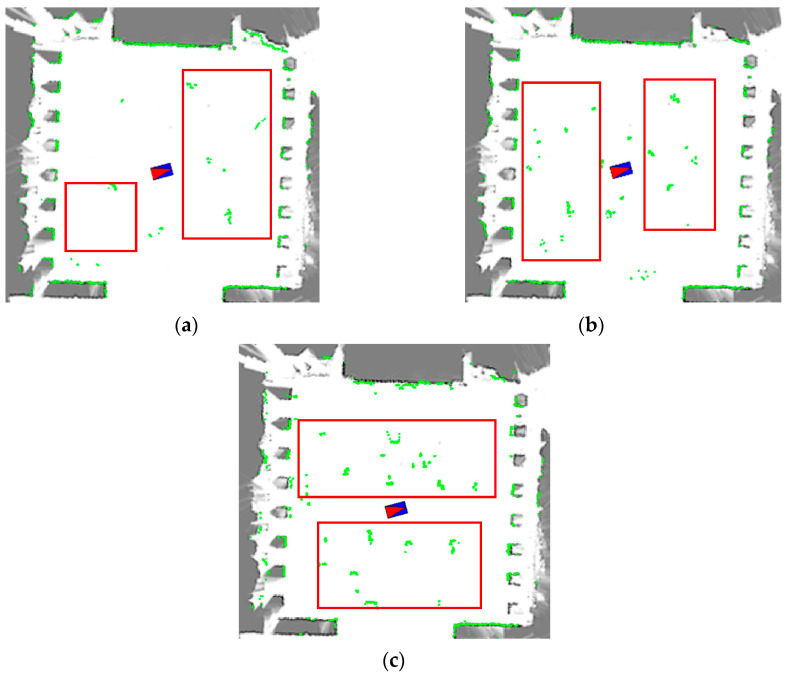
LiDAR measurement in different dynamic scenes at site 2. (**a**) Low dynamic environment. (**b**) Medium dynamic environment. (**c**) High dynamic environment.

To construct fuzzy logic rules for localization fluctuation estimation, 100 frames of LiDAR measurement are collected at each of the three sites in different dynamic scenarios using a mobile robot. In total, 900 frames of LiDAR data were provided for the fuzzy estimation. The LiDAR data from low to high dynamic scenes at Site 2 in the square are shown in Figure 3a. The LiDAR data from low to high dynamic scenes at Site 2 in the square are shown in Figure 1. Specifically, as shown in Figure 1, the low dynamic scenes have less than 10 percent of the observed noise, which is ideal for localization. The medium dynamic scenario is depicted in Figure 3b, where the observed noise accounts for 20 to 30 percent of the overall observed data, a common and realistic scenario. Extremely dynamic scenarios, such as the one shown in Figure 3c, will have noise levels close to 50 percent, which can easily lead to localization fluctuations or even localization failure.

Next, we determine the relationship between $V_{p}$ and E, and the mapping between $E_{p}$ and E. We put a different number of obstacles around the robot and record 100 localization results. $V_{i}$ and E correspond to each localization result at three sites. In this way, as indicated in Table 1, we get the $V_{i}$ and E of 100 localization results at three sites in different dynamic environments. And, as the dynamic level of the scene increases, $V_{i}$ and E become correspondingly larger.

According to the $V_{i}$ and E in different dynamic scenes, we can know the status of the localization fluctuations to which $V_{p}$ and E belong. Meanwhile, for example, when *i = x*, Equation (10) can be set to

(33) $$f_{Vx}(V_{x})=\left\{\begin{array}{cc} 0.00 & 0.00\leq V_{x}<1.93\times 10^{-4}\\ 0.25 & 1.93\times 10^{-4}\leq V_{x}<3.23\times 10^{-4}\\ 0.5 & 3.23\times 10^{-4}\leq V_{x} \end{array}\right.,\;f_{E}(E)=\left\{\begin{array}{cc} 0 & 0.00\leq E<6.37\\ 0.25 & 6.37\leq E<6.47\\ 0.5 & 6.47\leq E \end{array}\right.$$

Specifically, the relationship between $V_{pi}$ and $V_{i}$, and the mapping between $E_{p}$ and E, are shown in Table 2. We have grouped $V_{pi}$ and $E_{p}$, which fit into different fluctuation ranges, into one category. The 300 $V_{pi}$ and $V_{i}$ ranges are linked. Similarly, the mapping of the ranges of E and $E_{p}$ is found. 

**Figure 4:**
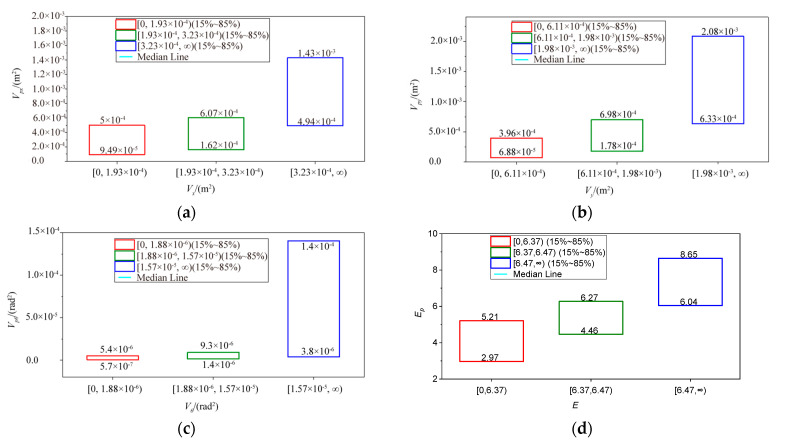
The distribution of $V_{p}$ and $E_{p}$ in the different ranges of V and E. (**a**) $V_{px}$ in the different ranges of $V_{x}$. (**b**) $V_{py}$ in the different ranges of $V_{y}$. (**c**) $V_{p\theta }$ in the different ranges of $V_{\theta }$. (**d**) $E_{p}$ in the different ranges of E.

The distribution of $V_{pi}$ and $E_{p}$ in the different ranges of $V_{i}$ and E are shown in Figure 4. We have used box plots to illustrate the distribution characteristics. It is easy to see that there are overlapping parts of $V_{pi}$ and $E_{p}$ in different ranges, which provide for the construction of fuzzy rules. According to Figure 4, the boxes in different ranges have a distinct overlap. Hence, we can easily choose the fuzzy partition boundary $V_{pi1}□V_{pi4}$ and $E_{p1}□E_{p4}$ for $V_{pi}$ and $E_{p}$, respectively. Finally, the “premise” of the fuzzy rule base defines $V_{pi}$ and $E_{p}$, and the fluctuation values $f_{Vi1}□f_{Vi3}$ and $f_{E1}□f_{E3}$ are the “conclusion”. Equations (13) and (14), when *i* = *x*, can be rewritten as

(34) $$\begin{array}{ccccc} \mathrm{rule}\;1:\;if & 0.00\leq V_{px}\leq 5\times 10^{-4} & then\;f_{Vx1}=f_{Vx}(V_{x}) & s.t. & 0.00\leq V_{x}<1.93\times 10^{-4}\\ \mathrm{rule}\;2:\;if & 1.62\times 10^{-4}\leq V_{px}\leq 6.07\times 10^{-4} & then\;f_{Vi2}=f_{Vx}(V_{x}) & s.t. & 1.93\times 10^{-4}\leq V_{x}<3.23\times 10^{-4}\\ \mathrm{rule}\;3:\;if & 4.94\times 10^{-4}\leq V_{px} & then\;f_{Vi3}=f_{Vi}(V_{x}) & s.t. & 3.23\times 10^{-4}\leq V_{x} \end{array}$$

(35) $$\begin{array}{ccccc} \mathrm{rule}\;1:\;if & 0.00\leq E_{p}\leq 5.21 & then\;f_{E1}=f_{E}(E) & s.t. & 0.00\leq E<6.37\\ \mathrm{rule}\;2:\;if & 4.46\leq E_{p}\leq 6.27 & then\;f_{E2}=f_{E}(E) & s.t. & 6.37\leq E<6.47\\ \mathrm{rule}\;3:\;if & 6.04\leq E_{p} & then\;f_{E3}=f_{E}(E) & s.t. & 6.47\leq E \end{array}$$

#### 6.2.2. Experimental Results of Adaptive MPC

**Figure 5:**
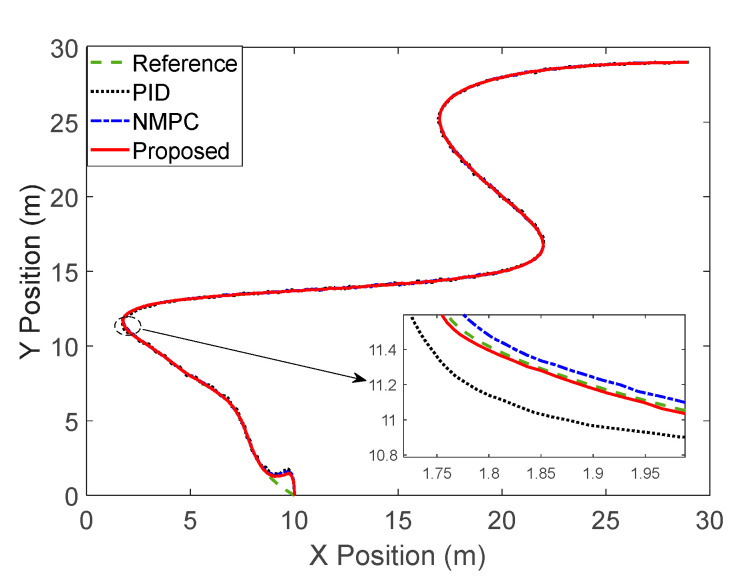
Tracking response of the comparison method.

Furthermore, to verify the superiority of the control method considering location uncertainty, we choose the following comparison methods: (1) The traditional PID control method with optimizing parameters, where $K_{p}=0.5$, $K_{i}=1.5$ and $K_{d}=0.1$. (2) The proposed control method does not consider the localization fluctuation (NMPC) with $N_{c}=N_{p}=5$. In the proposed control method, the control parameters are set as follows: $k_{p}=k_{c}=5$, $k_{1}=k_{2}=10$. The specific tracking process of the comparison method is described as follows:

The trajectory tracking of various comparison methods is shown in Figure 5. The initial point of the four-wheel differential mobile robot is (10, 0). It can be seen from Figure 5 that there is an error between the attitude angle of the initial point and the slope of the curve, resulting in a large tracking error. With time adjustment, all control methods can achieve fast convergence. It can be seen from the enlarged drawing of Figure 5 that compared with the proposed method and NMPC method, PID has a large oscillation. Further analysis shows that the proposed method has a better tracking effect.

**Figure 6:**
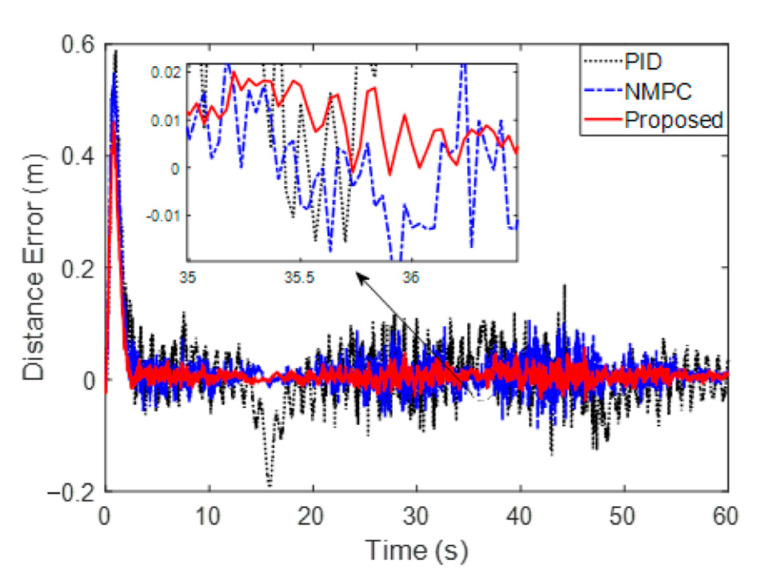
Distance error of the comparison method.

**Figure 7:**
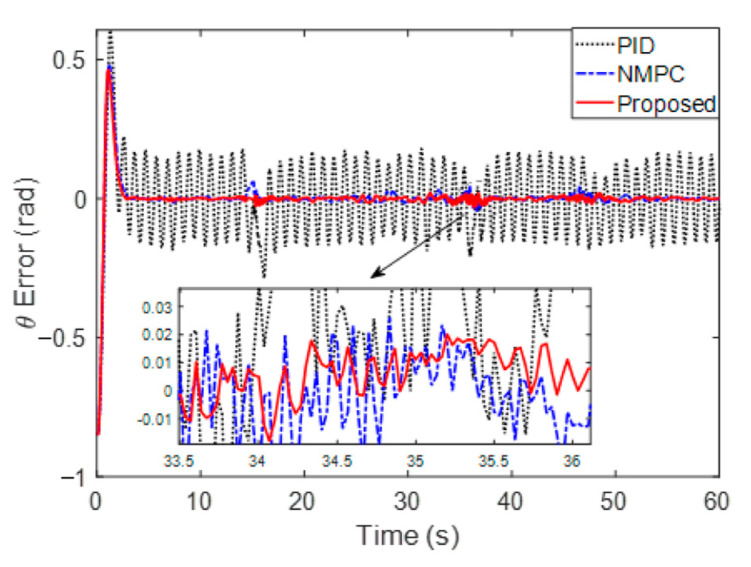
Orientation error of the comparison method.

Figure 6 and Figure 7 are the distance error and angle error, respectively. Specifically, Figure 7 shows that the overshoots of the PID and NMPC are 0.5903 m and 0.5502 m, while the proposed method is 0.4603 m. Furthermore, by comparing the proposed method with NMPC, it is found that the tracking process is optimized through adaptive step adjustment. On closer inspection, the average errors in the stable stage (10 s~60 s) of PID, NMPC and proposed methods are 0.0374 m, 0.0177 m and 0.0096 m, which show that the errors are reduced by 74.3% and 45.8%, respectively. It is found in 0 that the angle error of the PID method fluctuates greatly, while the NMPC and the proposed method will be limited to a small range. The average angle errors of PID and NMPC are 0.1001 rad and 0.0062 rad during a 10~60 s stabilization period, while the proposed method with a small error is 0.0047 rad. The stable errors are reduced up to 95.3% and 31.9%, respectively. By comparison, the proposed method achieves better error suppression. 

**Figure 8:**
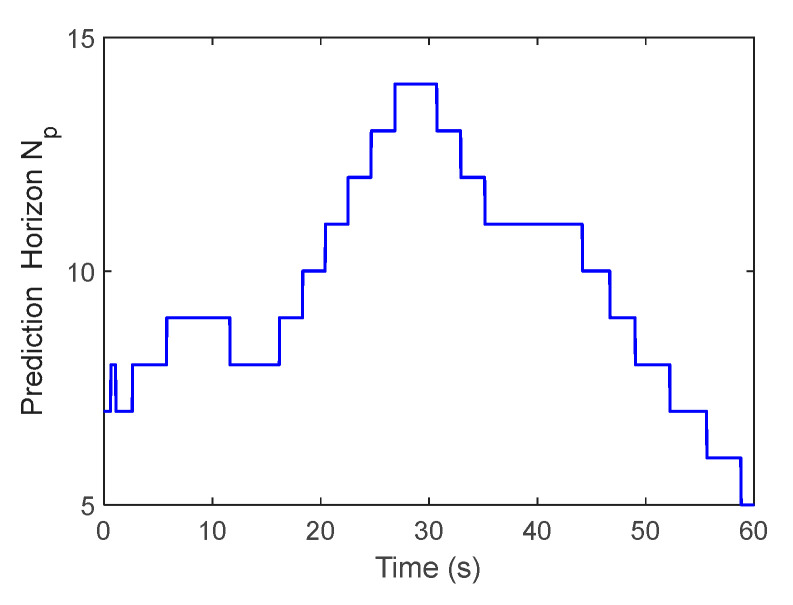
Adjustment of the prediction horizon.

From Figure 8, the adjustment of the prediction horizon is given through the localization fluctuation output by the localization module to ensure the control’s stability and accuracy under the high localization fluctuation scenario. Therefore, compared with the traditional PID and NMPC methods, the proposed method can realize the dynamic adjustment of the step size, improve tracking accuracy and reduce motion fluctuation.

## 7. Conclusions and Outlook

In this paper, an adaptive MPC for mobile robots with the fuzzy estimation of localization fluctuations was discussed, which accurately evaluates the localization state and effectively improves control accuracy and robustness. First of all, a localization fluctuation estimation based on fuzzy logic rules was proposed, which takes into account both the effects of variance and information entropy, thereby obtaining accurate estimates of localization fluctuations. Then, a modified kinematics model with external disturbance using the Taylor expansion-based linearization was constructed. In addition, according to localization fluctuation, an enhanced MPC with an adaptive adjustment of predictive step size was proposed to maintain the stability of the control system in dynamic scenes. The experimental results show that the step size of MPC can be effectively and adaptively adjusted with localization fluctuation in different dynamic scenes, avoiding the generation of oscillation. In the stable tracking phase, the proposed method reduced the tracking distance error by 74.3% and 45.8% compared with PID and NMPC. In addition, in comparison to PID and NMPC, the tracking angle errors of the proposed method decreased by 95.3% and 31.9%, respectively. In future work, we will explore the potential of deep learning for applications in localization fluctuation assessments and improve the control framework to better integrate localization fluctuation information.

## Figures and Tables

**Table 1:** The results of the $V_{i}$ and E of multiple localization results in different dynamic environments.

		Low Dynamic Scene				Medium Dynamic Scene				High Dynamic Scene			
		*V_x_*/(m^2^)	*V_y_*/(m^2^)	*V_θ_*/(rad^2^)	*E*	*V_x_*/(m^2^)	*V_y_*(m^2^)	*V_θ_*/(rad^2^)	*E*	*V_x_*/(m^2^)	*V_y_*(m^2^)	*V_θ_*/(rad^2^)	*E*
Site 1		1.24 × 10^−4^	3.23 × 10^−4^	1.53 × 10^−6^	6.33	2.19 × 10^−4^	8.55 × 10^−4^	1.93 × 10^−6^	6.41	4.67 × 10^−4^	6.28 × 10^−3^	7.48 × 10^−5^	6.56
Site 2		1.62 × 10^−4^	5.71 × 10^−4^	5.57 × 10^−7^	6.32	2.09 × 10^−4^	7.45 × 10^−4^	1.04 × 10^−6^	6.43	4.33 × 10^−4^	1.26 × 10^−3^	2.70 × 10^−6^	6.51
Site 3		1.80 × 10^−4^	2.63 × 10^−4^	1.48 × 10^−6^	6.37	2.66 × 10^−4^	9.09 × 10^−4^	4.76 × 10^−6^	6.39	3.45 × 10^−4^	1.83 × 10^−3^	8.97 × 10^−6^	6.54

**Table 2:** The results of $V_{pi}$ and $E_{p}$ in different ranges.

Range	Range1: $0.00\leq V_{x}<1.93\times 10^{-4}$			Range2: $1.93\times 10^{-4}\leq V_{x}<3.23\times 10^{-4}$			Range3: $3.23\times 10^{-4}\leq V_{x}$		
$V_{px}$/(m^2^)	7.03 × 10^−4^	3.06 × 10^−4^	5.05 × 10^−4^	1.17 × 10^−2^	3.54 × 10^−4^	3.92 × 10^−4^	1.18 × 10^−1^	9.10 × 10^−4^	5.84 × 10^−4^
	98 lines of $V_{px}$								
	4.76 × 10^−4^	3.37 × 10^−4^	4.57 × 10^−4^	3.96 × 10^−4^	2.88 × 10^−4^	1.49 × 10^−4^	1.39 × 10^−3^	7.85 × 10^−4^	8.83 × 10^−4^
Range	range1: $0.00\leq V_{y}<6.11\times 10^{-4}$			range2: $6.11\times 10^{-4}\leq V_{y}<1.98\times 10^{-3}$			range3: $1.98\times 10^{-3}\leq V_{y}$		
$V_{py}$/(m^2^)	8.01 × 10^−4^	4.08 × 10^−4^	1.45 × 10^−4^	2.61 × 10^−2^	3.24 × 10^−4^	9.01 × 10^−4^	2.82 × 10^−1^	1.20 × 10^−2^	1.37 × 10^−3^
	98 lines of $V_{px}$								
	2.75 × 10^−4^	1.86 × 10^−4^	2.69 × 10^−4^	2.18 × 10^−4^	3.21 × 10^−4^	1.40 × 10^−4^	1.12 × 10^−3^	8.20 × 10^−4^	1.14 × 10^−3^
Range	range1: $0.00\leq V_{\theta }<1.88\times 10^{-6}$			range2: $1.88\times 10^{-6}\leq V_{\theta }<1.57\times 10^{-5}$			range3: $1.57\times 10^{-5}\leq V_{\theta }$		
$V_{p\theta }$/(rad^2^)	4.59 × 10^−6^	1.03 × 10^−6^	3.50 × 10^−7^	3.14 × 10^−4^	8.30 × 10^−7^	9.34 × 10^−7^	5.13 × 10^−3^	1.20 × 10^−4^	1.99 × 10^−5^
	98 lines of $V_{p\theta }$								
	7.66 × 10^−6^	2.45 × 10^−6^	3.01 × 10^−6^	9.88 × 10^−7^	2.12 × 10^−6^	1.69 × 10^−6^	1.09 × 10^−4^	2.11 × 10^−5^	5.95 × 10^−5^
Range	range1: 0.00 ≤ *E* <6.37			range2: 6.37 ≤ *E* <6.47			range3: 6.47 ≤ *E*		
$E_{p}$	6.22	2.51	5.18	5.05	6.30	5.00	1.11	8.85	7.79
	98 lines of $E_{p}$								
	4.88	5.03	4.07	5.73	5.39	5.19	7.29	7.98	7.94

## Data Availability

Not applicable.

## Notations and Abbreviations

The following key notations and abbreviations are used in this paper.

**Notations/Abbreviations**	**Descriptions**
SMC	sliding mode control
MPC	model predictive control
J(⋅)	cost function
$N_{p},N_{c}$	Prediction/control horizon
P,R	weight matrix
v	linear velocity
ω	angular velocity
$f_{\chi_{r}}(\cdot )$,$f_{u_{r}}(\cdot )$	coefficient matrices
O(r)	higher order remainder of the Taylor expansion
$P_{l}$	estimated localization fluctuation value
$k_{1,2}$	adjustment coefficient
$k_{c},k_{p}\in N_{+}$	minimum adjustment coefficient
